# Isolation and Characterization of a Thermophilic, Obligately Anaerobic and Heterotrophic Marine *Chloroflexi* Bacterium from a *Chloroflexi*-dominated Microbial Community Associated with a Japanese Shallow Hydrothermal System, and Proposal for *Thermomarinilinea lacunofontalis* gen. nov., sp. nov.

**DOI:** 10.1264/jsme2.ME12193

**Published:** 2013-05-11

**Authors:** Takuro Nunoura, Miho Hirai, Masayuki Miyazaki, Hiromi Kazama, Hiroko Makita, Hisako Hirayama, Yasuo Furushima, Hiroyuki Yamamoto, Hiroyuki Imachi, Ken Takai

**Affiliations:** 1 Subsurface Geobiology & Advanced Research (SUGAR) Project, Extremobiosphere Research Program, Institute of Biogeosciences, Japan Agency for Marine-Earth Science & Technology (JAMSTEC), 2–15 Natsushima-cho, Yokosuka 237–0061, Japan; 2 Marine Biodiversity Research Program, Institute of Biogeosciences, Japan Agency for Marine-Earth Science & Technology (JAMSTEC), 2–15 Natsushima-cho, Yokosuka 237–0061, Japan

**Keywords:** *Chloroflexi*, *Anaerolineae*, thermophile, hydrothermal, anaerobe

## Abstract

A novel marine thermophilic and heterotrophic *Anaerolineae* bacterium in the phylum *Chloroflexi*, strain SW7^T^, was isolated from an *in situ* colonization system deployed in the main hydrothermal vent of the Taketomi submarine hot spring field located on the southern part of Yaeyama Archipelago, Japan. The microbial community associated with the hydrothermal vent was predominated by thermophilic heterotrophs such as *Thermococcaceae* and *Anaerolineae*, and the next dominant population was thermophilic sulfur oxidizers. Both aerobic and anaerobic hydrogenotrophs including methanogens were detected as minor populations. During the culture-dependent viable count analysis in this study, an *Anaerolineae* strain SW7^T^ was isolated from an enrichment culture at a high dilution rate. Strain SW7^T^ was an obligately anaerobic heterotroph that grew with fermentation and had non-motile thin rods 3.5–16.5 μm in length and 0.2 μm in width constituting multicellular filaments. Growth was observed between 37–65°C (optimum 60°C), pH 5.5–7.3 (optimum pH 6.0), and 0.5–3.5% (w/v) NaCl concentration (optimum 1.0%). Based on the physiological and phylogenetic features of a new isolate, we propose a new species representing a novel genus *Thermomarinilinea*: the type strain of *Thermomarinilinea lacunofontalis* sp. nov., is SW7^T^ (=JCM15506^T^=KCTC5908^T^).

Distribution of the putative non-phototrophic *Chloroflexi* class *Anaerolineae* has been revealed by culture-independent analyses in diverse marine, terrestrial and artificial environments including hydrothermal environments, and it is known to be one of the uncultivated bacterial lineages (28, 32). In this decade, 9 species, *Anaerolinea thermophila*, *Anaerolinea thermolimosa*, *Levilinea saccharolytica*, *Leptolinea tardivitalis*, *Bellilinea caldifistulae*, *Longilinea arvoryzae*, *Thermanaerothrix daxensis* and *Ornatilinea apprima*, have been isolated (6, 21, 32). The members of *A. thermophila*, *A. thermolimosa*, *L. saccharolytica*, *L. tardivitalis* and *B. caldifistulae* were isolated from methanogenic sludges under mesophilic or thermophilic conditions. In contrast to these species, *L. arvoryzae* was obtained from a rice field soil, and two species of *T. daxensis* and *O. apprima* were derived from terrestrial subsurface hot aquifers (6, 21, 31). Furthermore, isolation of two marine strains from an enrichment reactor of subseafloor sediments was reported recently (10). These species have similar physiological characteristics, such as being obligately anaerobic, mesophilic to moderately thermophilic, and multicellular filamentous heterotrophs utilizing carbohydrates and amino acids (or peptides). All the *Anaerolineae* species except for *O. apprima* are slowly growing microorganisms with generation times of 10–100 hours under the optimum growth conditions (6, 21, 32).

On the other hand, no *Anaerolineae* isolates have been reported from hydrothermal environments although the class has been recognized as one of the significant bacterial populations associated with these environments based on SSU rRNA gene clone analyses (28). The relatively slow growth of this group, and co-occurrence of diverse heterotrophic bacteria in hydrothermal vent environments might have prevented enrichment and isolation of the *Anaerolineae* species. In hydrothermal environments, the predominance of thermophilic and heterotrophic *Thermococcaceae* archaea and other thermophilic heterotrophs growing faster than *Anaerolineae* species has likely inhibited the enrichment and isolation of *Anaerolineae* species. We fortunately isolated the marine thermophilic strain of the *Anaerolineae* from a shallow submarine hydrothermal field in the southern part of the Yaeyama Archipelago, Japan during culture-dependent viable counting analysis of the microbial ecosystem associated with hydrothermal activity. We report here the microbial community structure attached to an *in situ* colonization system (ISCS) deployed in the main hydrothermal vent of the hydrothermal field, which was the isolation source of the *Anaerolineae* strain. Furthermore, we describe the physiological and partial chemotaxonomic characterization of a novel strain belonging to the class *Anaerolineae*.

## Materials and Methods

### Sample collection

Taketomi submarine hot spring shallow hydrothermal field (24° 20′ 9″N, 124° 06′ 10″E) is located in a coral lagoon in the southern part of the Yaeyama Archipelago, Japan (4, 15). The geochemistry of venting fluids, microbial mat formation in the hydrothermal environments, and diverse novel bacterial strains have already been reported (8, 9, 16, 27). An in situ colonization system with a self-recording temperature probe (STR-ISCS) (25) was set in the main vent emission for 5 days. Pumiceous materials were placed in the ISCS to support microbial attachment (Fig. S1). Placement and retrieval of the STR-ISCS were conducted by scuba divers (June 2006). The temperature of the main vent emission in this hydrothermal field determined by the STR-ISCS was a constant 52°C at a water depth of 17 m. The retrieved pumice in the STR-ISCS was sub-sampled for cultivation and stored anaerobically (with 0.05% neutralized Na_2_S) in a Schott glass bottle under 100% N_2_ (100 kPa), sealed with butyl rubber stoppers. Samples were stored at 5°C. The sample for molecular analyses was stored at −80°C.

### Enrichment and purification

The abundance of viable archaea and bacteria represented by a variety of physiological and metabolic characteristics was determined by a series of serial dilution cultures under various cultivation conditions. In this study, cultures were grown in a 15 mL test tube containing 3 mL medium. MJYPS medium (29) was used at 85 and 55°C for thermophilic to hyperthermophilic fermentative sulfur-reducing heterotrophs, MMJ medium (26) was used at 70°C for thermophilic methanogens, MMJSO medium (20) was used at 85, 70 and 55°C for thermophilic to hyperthermophilic sulfate reducers, MMJS medium (18) was used at 85 and 70°C for thermophilic to hyperthermophilic sulfur reducers, MMJHS medium (25) with three types of head space gases of 80% H_2_ and 20% CO_2_ (200 kPa), and 79% H_2_, 20% CO_2_ and 1% O_2_ (200 kPa) was used at 85, 70 and 55°C for thermophilic to hyperthermophilic, anaerobic to microaerophilic autotrophs (nitrate-reducing and microaerophilic hydrogen oxidizers and sulfur oxidizers), and MMJYPS medium (19) was used at 70 and 55°C for strictly anaerobic thermophilic mixotrophs. Compositions of these media are summarized in the supplementary material. The microorganisms present in the most diluted series of the medium at each temperature were isolated by the subsequent extinction–dilution method (29).

In order to obtain an isolate of thermophilic *Anaerolineae* strain, MJYPS medium was used for serial dilution cultivation at 55°C. Since the growth of a potential strain was unstable, modified MJY (MJY medium supplemented with 0.1% NaHCO_3_) medium under head space gas of N_2_ and CO_2_ mixture (16) was used for further isolation and characterization. MJY medium consists of MJ synthetic seawater with 0.1% yeast extract. The medium was prepared as follows: 1) Modified MJY medium with resazurin was autoclaved under N_2_ gas; 2) The medium was pressurized with N_2_/CO_2_ gas mixture (80:20) at 150 kPa; 3) Neutralized Na_2_S solution (final 0.05%) was added to the medium. Pure culture was obtained by the dilution to extinction technique. The first dilution to extinction was carried out at 45°C, and the following dilution to extinction was conducted at 65°C. Purity of the isolate was tested by microscopic observation for cultures obtained at different growth temperatures (30–70°C) with MJY, repeating SSU rRNA gene direct sequencing and SSU rRNA gene clone analysis described below. In addition, the absence of *Thermococcaceae* in the culture was also confirmed by a cultivation test using MJYPS medium at 70°C.

### Microscopic observations

Cells were routinely observed using an Olympus BX51 microscope (Tokyo, Japan). Scanning electron microscope observation was carried out using JSM-6700F (JEOL, Tokyo, Japan) as described previously (3). Transmission electron micrographs of negatively stained cells and thin cell sections were obtained as described by Zillig *et al.* (34). Cells grown in modified MJY medium at 60°C in the late exponential phase were used for transmission electron microscope observations using JEOL JEM-1210 at an accelerating voltage of 80 kV.

### Nucleic acid analyses

Environmental DNA was extracted using the Ultra Clean Soil DNA Purification Mega Kit (Mo Bio Laboratories, Solana Beach, CA, USA). The archaeal and bacterial SSU rRNA genes were amplified from the DNA assemblage using LA *Taq* polymerase with GC buffer (Takara Bio, Otsu, Japan) with primer sets of Arch21F-U907R and B27F-U907R, respectively (2, 11) (Table S1). Gene fragments of *mcrA* were also obtained with a primer set of ME3MF and ME2r’ (7, 17) using SYBR Premix Ex *Taq* II (Takara Bio). The DNA amplification conditions are summarized in Table S1. PCR amplification was performed using a thermal cycler GeneAmp 9700 (Perkin-Elmer, Waltham, MA, USA). The amplified gene fragments were cloned into pCR2.1 vector (Invitrogen, Carlsbad, CA, USA) and were sequenced with M13 primer by the deoxynucleotide chain-termination method with a DNA sequencer model 3130XL (Applied Biosystems, Carlsbad, CA, USA).

The archaeal and prokaryotic SSU rRNA genes were quantified according to a previously published method (24) with minor modifications using the 7500 Real Time PCR System (Applied Biosystems) (17). Sets of primers and probes used for archaeal and total prokaryotic SSU rRNA genes were Arch349F-516F-806R and Uni340F-516F-806R, respectively, and PCR conditions are summarized in Table S1. Quantification of *mcrA* with a primer set of ME3MF and ME2r’ was also conducted using SYBR Premix Ex *Taq* II as described previously (17). Abundance of each gene was determined as an average of duplicate or triplicate analyses.

Genomic DNA for PCR amplification from isolates was purified using the Illustra bacteria genomicPrep Mini Spin Kit (GE Healthcare, Buckinghamshire, England). The SSU rRNA gene was amplified by PCR using LA *Taq* polymerase with GC buffer. Primers Bac27F and 1492R (2, 11) were used for PCR amplification (Table S1). Amplified SSU rRNA gene fragment served as a direct template for sequencing analysis.

In order to know the phylogenetic positions of the SSU rRNA genes from the environmental DNA and isolates, these sequences were analyzed by the Blast search program in DDBJ, and ARB software (14). Alignments of SSU rRNA gene sequences from the *Chloroflexi* species, strain SW7^T^ and environmental DNA were generated using ARB software (14), and the SSU rRNA gene trees using unambiguous residues were constructed by the PhyML method using SeaView4 software (5). Alignment of *mcrA* sequences and the neighbor-joining phylogenetic tree using unambiguous nucleic acid residues were constructed by Clustal X ver 2.0 (12).

The G+C content of genomic DNA from a *Chloroflexi* strain SW7^T^ prepared as in Lauer *et al.* (1986) (13) was determined by direct analysis of deoxyribonucleotides by HPLC (30).

### Growth characteristics of *Chloroflexi* strain

Growth of the isolate was determined by microscopic observation in most cases. Utilization of possible electron donors (H_2_ and yeast extract) and acceptors (sulfur (0.3 w/v%), thiosulfate (0.1%), sulfate (0.3%), sulfite (0.03%), l-cystine (0.03%), glutathione (oxidized form) (0.03%), nitrate (0.1%), nitrite (0.03%)) was examined with modified MJY medium. Cultures were grown in a 15 mL test tube containing 3 mL medium during the tests for growth characteristics, except for the experiments that determined the growth rates described below.

To determine the growth range of temperature, NaCl concentration and pH, cultures were grown in a 100 mL serum bottle containing 33 mL medium with the head-space (N_2_/CO_2_) gas described above in temperature control drying ovens. Modified MJY medium was used for cultivation tests for temperature (30–70°C) and NaCl concentration (0–5%). The effect of initial pH on growth was examined at 60°C using modified MJY medium. Various pHs (pH 5–8) were adjusted by changing NaHCO_3_ concentrations in the MJY medium (0 or 0.1%), CO_2_ concentrations in head-space gas (0–20%) and the pressure of head-space gas (100–170 kPa). Aggregate formation and precipitation of medium inhibited cell counting and the protein concentration measurement, respectively. Thus, growth rate was determined from the cellular ATP concentration with respective intervals as follows. Cells in 1 mL culture were filtrated through a 0.2 μm polycarbonate filter (12 mm in diameter), and cellular ATP concentration was measured using ATP analyzer AF-100 (TOADKK, Tokyo, Japan) following the manufacturer’s instructions. ATP concentration was measured in duplicate.

The utilization of carbon sources was tested using MJY medium without yeast extract supplemented with the vitamin mixture (1) and the following carbon sources at 60°C: yeast extract, tryptone, peptone, Casamino acids (Difco), gelatin, chitin, starch, glucose, fructose, maltose, galactose, lactose, cellobiose, xylose, sucrose, rhamnose, mannose, ethanol, methanol, glycerol, acetate, pro-pionate, pyruvate, formate, fumarate, citrate, malate, succinate, tartarate, glutamate, glycine, alanine and xylan (each substrate was tested at 2 concentrations; 0.02% and 0.1%). The substrate utilization test with 0.01% yeast extract was also performed for substrates that did not support growth as a sole carbon source in the absence of yeast extract. Head-space gas (N_2_/CO_2_) was prepared as described above.

The sensitivity of antibiotics such as ampicillin, chloramphenicol, erythromycin, kanamycin, penicillin G, novobiocin, spectinomycin, tetracycline, streptomycin, vancomycin and rifampicin at 50 μg mL^−1^ was tested at 60°C using modified MJY medium.

### Fatty acid analysis of *Chloroflexi* strain

The cellular fatty acid composition of strain SW7^T^ was analyzed with cells grown in modified MJY medium at 60°C in the late exponential phase. Lyophilized cells were suspended in 1 mL anhydrous methanolic HCl and heated at 100°C for 3 h. The fatty acid methyl esters (FAMEs) were extracted three times with n-hexane. Concentrated FAMEs were analyzed using a gas chromatography-mass spectrometer (Xcalibur for Trace DSQ; Thermo Fisher Scientific, Waltham, MA, USA).

### Nucleotide sequence accession numbers

The GenBank/EMBL/DDBJ accession number for the SSU rRNA gene sequence of *Chloroflexi* strain SW7^T^ is AB669272. Environmental SSU rRNA and *mcrA* gene sequences, and SSU rRNA genes from representative strains of viable populations are also deposited in the public database with accession numbers AB752310 to AB752343.

## Results

### Environmental SSU rRNA gene and *mcrA* community structures

**Fig. 1:**
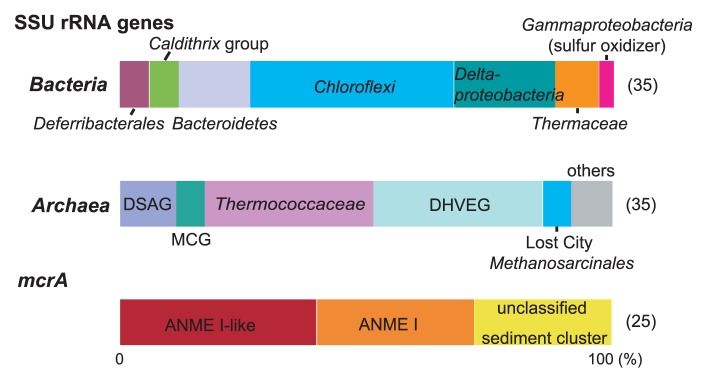
Bacterial and archaeal SSU rRNA genes and *mcrA* gene community structures obtained from the pumiceous materials in the ISCS deployed in the main hydrothermal vent of the Taketomi hydrothermal field. Numbers in parentheses indicate the number of clones sequenced.

Quantitative PCR analyses indicated that gene abundances of total prokaryotic SSU rRNA gene, archaeal SSU rRNA gene and *mcrA* were 2.4×10^9^, 4.8×10^7^ and 3.1×10^3^ copies g pumice in the ISCS^−1^, respectively. Compositions of bacterial and archaeal SSU rRNA gene and *mcrA* communities associated with the ISCS settled in the main vent emission are summarized in Fig. 1 and Table S2 and S3. Bacterial SSU rRNA gene community was predominated by *Chloroflexi* phylotypes belonged to *Anaerolineae* (Fig. S2), and deltaproteobacterial phylotypes were also detected as dominant populations. Except for one phylotype closely related to the gammaproteobacterial sulfur-oxidizing genus *Sulfurivirga*, other phylotypes detected in the bacterial SSU rRNA gene clone analysis were likely derived from heterotrophic bacterial populations. The archaeal SSU rRNA gene clone library was predominated by hyperthermophilic *Thermococcaceae* and the thermophilic Deep-sea Hydrothermal Vent Euryarchaeotic Group (DHVEG) represented by “*Candidatus* Acidulliprofundum” (22). Unusually, most of the *Themococcaceae* sequences represented by the phylotype TKM_WI_A50 were closely related to *Palaeococcus ferriphilus* (99.8% similarity) while *Thermococcus* or *Pyrococcus* species have been known to predominate in most of the hydrothermal vent ecosystems (28). One sequence grouped into methanogenic or methanotrophic Lost City *Methanosarcinales* was identified in the analysis. On the other hand, the *mcrA* community consisted of three phylogroups, ANME I, ANME I-like and unclassified sediment clusters, and this composition suggested that more than 70% of the *mcrA* sequences detected in this study derived from anaerobic methanotrophs (ANMEs) but not methanogens (Fig. 1, Fig S3, Table S3).

### Viable populations

**Fig. 2:**
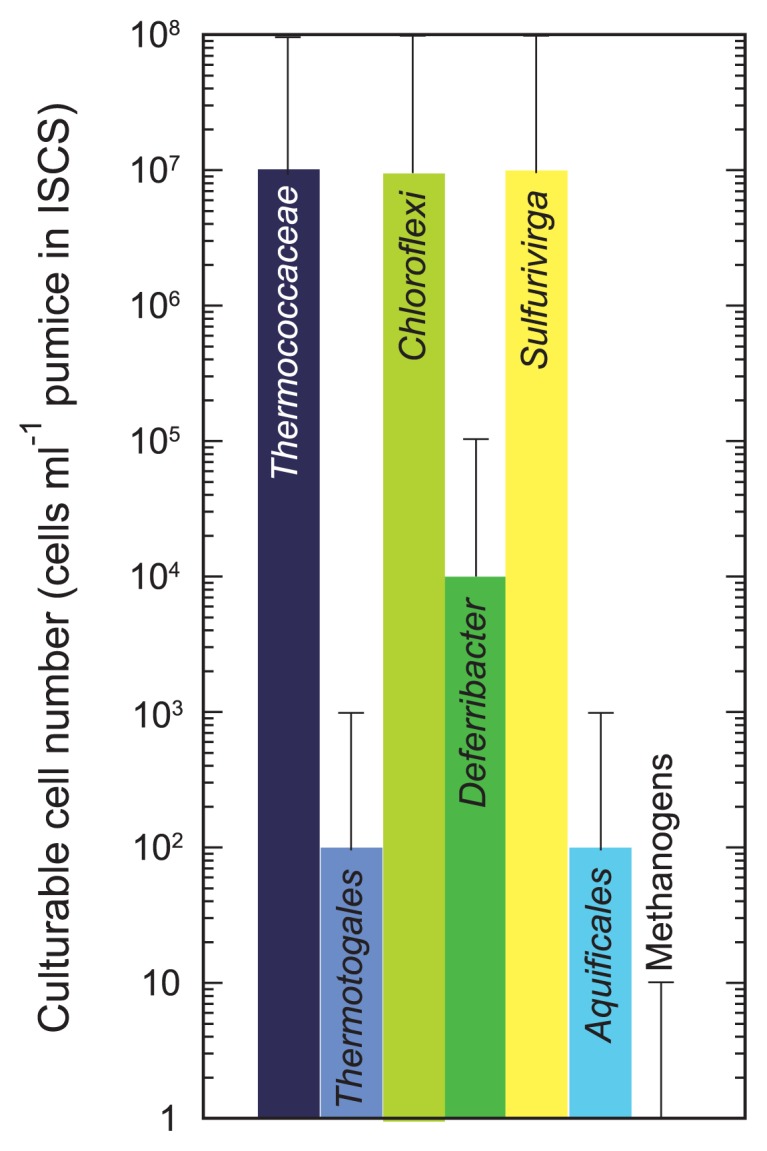
Viable microbial populations associated with the ISCS deployed in the main hydrothermal vent of the Taketomi hydrothermal field.

Heterotrophic *Thermococcaceae* and *Anaerolineae* members and chemolithoautotrophic sulfur-oxidizing gamma-proteobacterium *Sulfurivirga* sp. were found to be the most predominantly cultivated populations in the hydrothermal vent (Fig. 2, Table S4). Hydrogenotrophic viable populations of chemolithoautotrophic *Persephonella* sp. and mixotrophic *Deferribacter* sp. were obtained, although these viable numbers were more than 3 orders of magnitude smaller than those of fermentative heterotrophs and sulfur-oxidizing chemolithoautotrophs. Thermophilic methanogens and sulfate reducers were not detected.

The *Anaerolineae* member obtained in the viable count was phylogenetically distant from the previously isolated and/or characterized strains in this family. Thus, we tried to purify a strain and characterize it in detail, as described below.

### Purification and morphology of the *Anaerolineae* strain

From a diluted series of enrichment cultures at 55°C with MMJYS medium, into which pumiceous materials recovered from the STR-ISCS had been inoculated, an aggregate of non-motile thin rods was observed. Positive growth of the mono-morphotype was observed at the dilution rate of <10^−9^ cells mL^−1^ pumice without sulfide production, and no growth was observed at dilution rates of <10^−8^ cells mL^−1^ pumice (Fig. 2, Table S4). After several successive cultures, we determined a partial SSU rRNA gene sequence of the enrichment by direct sequencing of the PCR product without ambiguous residues. The result suggested that a single *Anaerolineae* species grew predominantly in the enrichment. Since the growth of the thin rods was unstable in the MMJYS medium and elemental sulfur was not reduced during culture, we examined several media and found that modified MJY medium could support stable growth of the thin rods. We then used the modified MJY medium for further purification and culture analyses. Since colony formation of the thin rods was not observed using a role-tube method, we applied the dilution to extinction technique serially for purification. Before purification using the serial dilution to extinction technique, we confirmed the contamination of hyperthermophilic *Thermococcaceae*-like and moderately thermophilic fermenters in the enrichment using MMJYPS and MJY medium incubated at 70 and 40°C, respectively, by microscopic observation; therefore, serial dilution to extinction was conducted at 40°C and then at 65°C to eliminate *Thermococcaceae*-like and moderately thermophilic fermenters, respectively. After purification, the absence of *Thermococcaceae*-like and moderately thermophilic fermenters was confirmed by the culture test as described above with no PCR amplification of the archaeal SSU rRNA gene. Purity of the isolate was also examined by sequencing the 5′ or 3′ end of 94 SSU rRNA gene fragments. Then, we determined the almost full length (1,435 bp) of the SSU rRNA gene sequence without ambiguous residues, and thus obtained strain SW7^T^ (=JCM 15506^T^=KCTC5908^T^).

**Fig. 3:**
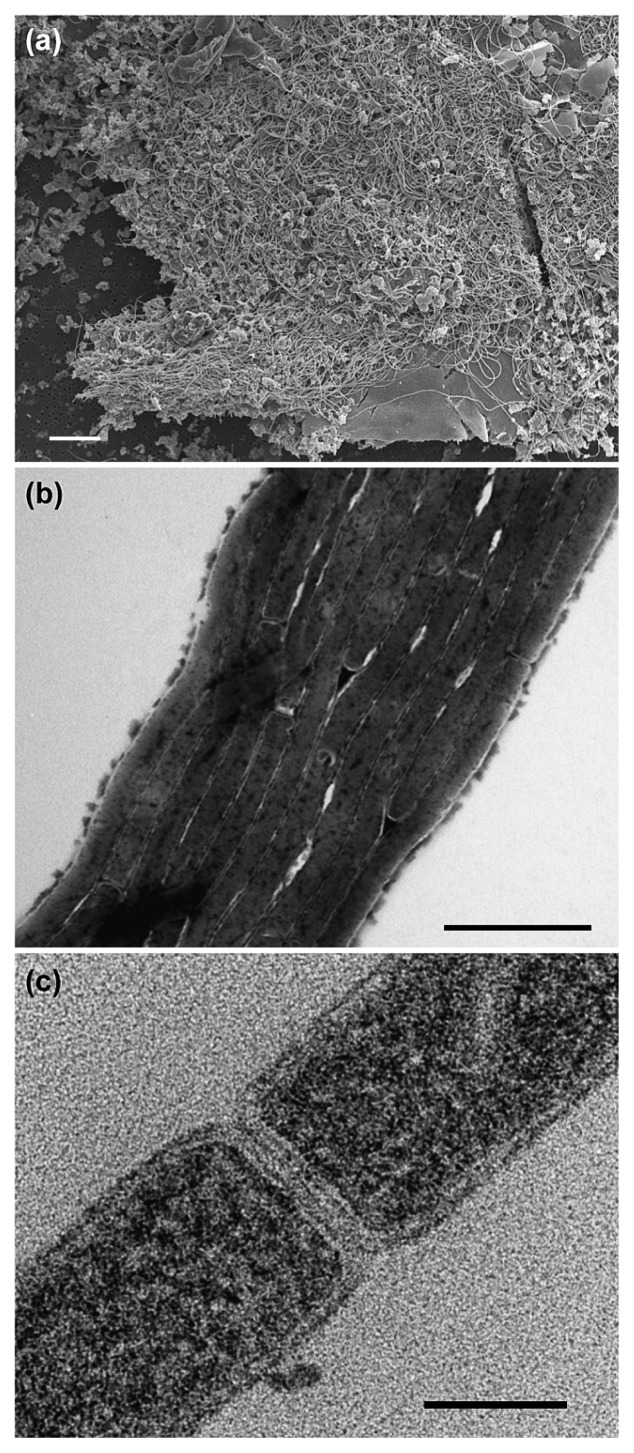
Scanning electron micrograph of SW7^T^ cells (a). Transmission electron micrographs of negatively stained (b) and thin section (c) cells of strain SW7^T^. Scale bars indicate 10 μm (a), 1 μm (b) and 0.1 μm (c).

Thin rod cells of strain SW7^T^ constituted multicellular filaments and formed large aggregates up to 1 cm in longest diameter (Fig. 3) at the bottom of the 15 mL test tube. Cells were about 3.5–16.5 μm in length, 0.2 μm in width without a flagellum and did not show gliding motility (Fig. 3). Spores were not found under any growth conditions or with any observation techniques.

### Growth characteristics

**Fig. 4:**
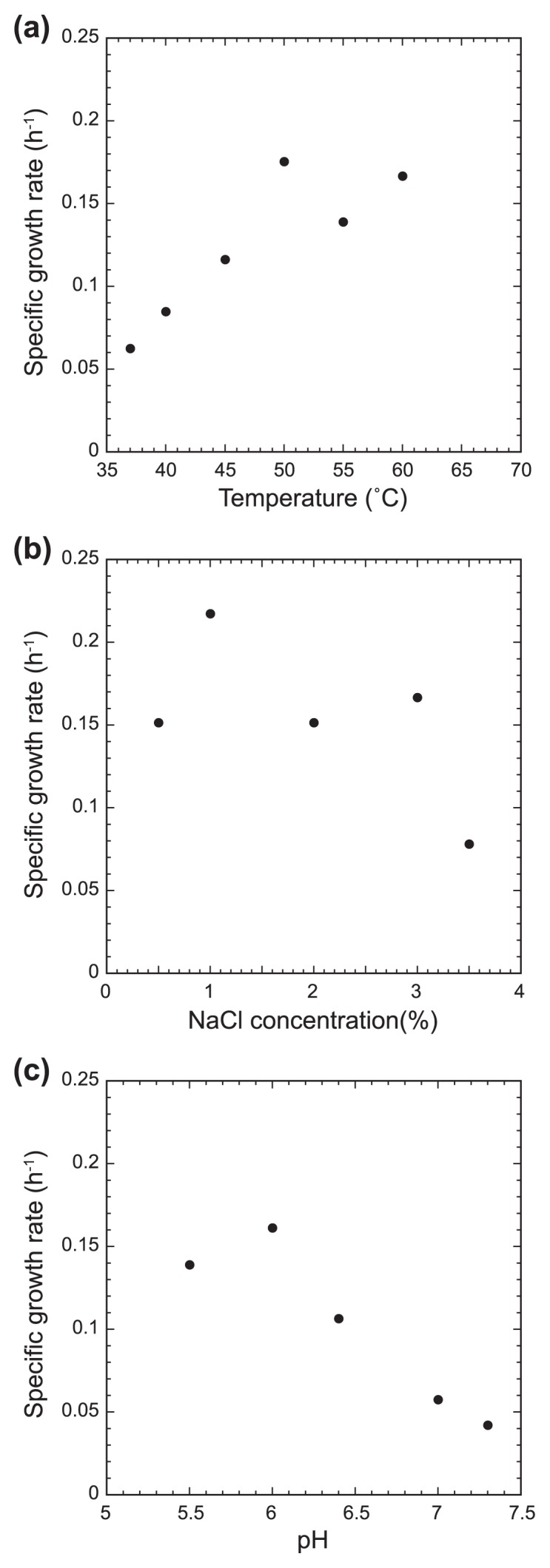
The effect of temperature (a), NaCl concentration (b) and (c) pH on growth of strain SW7^T^. Growth at 65°C was unstable and a reliable growth rate was not obtained. No growth occurred at 35 and 70°C, pH 5.0 and 7.6, and 0 and 4.0% NaCl concentration.

Strain SW7^T^ grew only by fermentation and did not utilize molecular oxygen (O_2_) or other potential electron acceptors. The isolate was sensitive to O_2_ and could not grow in the medium without reducing regents. Strain SW7^T^ grew in modified MJY medium over a temperature range of 37–65°C (optimum; 50–60°C) (Fig. 4). Growth was observed at 37–65°C, but growth at 65°C was unstable and sometimes stopped. No growth was observed at 30 and 70°C. The growth pH range was 5.5–7.3 (optimum pH was 6.0), and no growth occurred at pH 5.0 and 7.6. Effect of NaCl concentration on growth in modified MJY medium was tested, and growth was observed 0.5–3.5% (w/v) NaCl concentration (optimum 1.0%) (Fig. 4). The fastest doubling time was 4.6 h, observed at 60°C in modified MJY medium with 1.0% NaCl concentration. In the substrate utilization test, tryptone, peptone, casein, gelatin, chitin, glutamate, alanine, mannitol and citrate supported growth in the absence of yeast extract. Substrate utilization in the presence of 0.01% yeast extract was also tested only for substrates that were not utilized as a sole carbon source in the absence of yeast extract, but no substrates supported the growth of the strain.

The sensitivity of antibiotics was tested at 60°C using modified MJY medium. Growth was inhibited by chloramphenicol, erythromycin, novobiocin, spectinomycin and vancomycin at 50 μg mL^−1^. Strain SW7^T^ was insensitive to ampicillin, penicillin G, kanamycin, streptomycin, tetracycline and rifampicin at 50 μg mL^−1^.

### SSU rRNA gene phylogenetic analysis

An almost complete SSU rRNA gene sequence (1,435 bp) was obtained after repeating isolation and was analyzed by FASTA algorithm in DDBJ or EMBL. Similar sequences from characterized species to strain SW7^T^ belonged to the class *Anaerolineae* in the *Chloroflexi* subphylum I such as *Thermanaerothrix dexensis* (88.7%), *Levilinea saccharolytica* (88.2%), *Ornatilinea apprima* (88.2%), *Bellilinea caldifistulae* (87.3%), *Anaerolinea thermophila* (87.4%), *Leptolinea tardivitalis* (87.1%), *Longilinea arvoryzae* (87.3%) and *Anaerolinea thermolimosa* (86.9%). The SSU rRNA gene similarity value between the SW7^T^ and *Caldilinea aerophila* representing the class *Caldilineae* was 82.5%. Similarity values between SW7^T^ and environmental SSU rRNA gene sequences were also lower than 90% (data not shown).

**Fig. 5:**
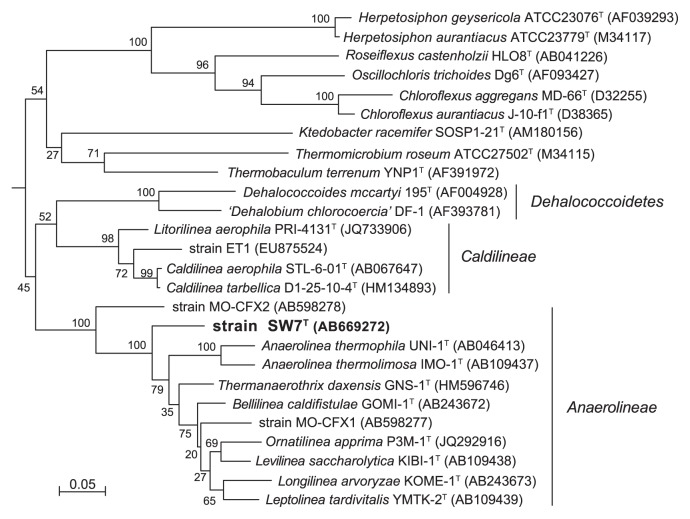
Phylogenetic tree of the phylum *Chloroflexi* based on SSU rRNA gene sequence by PhyML using 1,173 homologous sequence positions for each organism. Numbers indicate bootstrap values from 100 trials. Numbers in parentheses are GenBank/EMBL/DDBJ accession number. Bar indicates 5 substitutions per 100 nucleotides. *Desulfovibrio vulgaris* DSM644 (M34399) was used as an out-group.

Phylogenetic analysis based on SSU rRNA gene sequences from cultivated *Chloroflexi* species indicate that SW7^T^ belongs to the class *Anaerolineae* (Fig. 5). In the phylogenetic tree including environmental SSU rRNA gene sequences, strain SW7^T^ belonged to a cluster that was distant from branches including environmental *Anaerolineae* sequences obtained in this study (Fig. S2).

### Fatty acid composition and DNA base composition

The fatty acid composition of strain SW7^T^ was C_12:0_ (1.4%), C_16:0_ (47.0%), C_16:1_ (5.2%), C_18:0_ (37.3%) and C_18:1_ (9.1%). The abundance of C_18:0_ likely reflected the relatively higher growth temperature also observed in thermophilic *T. daxensis* but not detected in the mesophilic *Anaerolineae* species (21, 32). Unsaturated fatty acids only found in strain SW7^T^ were not observed in other *Aanerolineae* species (Table 1).

The DNA G+C content of strain SW7^T^ was 59.9 mol%, which is similar to *L. saccharolytica*, but 2–6 mol% higher than other *Anaerolineae* species. No significant relationship between the DNA G+C content and growth temperature was observed among the *Anaerolineae* species (Table 1).

## Discussion

### Microbial ecosystem of the hydrothermal environment

Both culture-dependent and -independent analyses showed that the microbial community associated with the hydrothermal vent was predominated by heterotrophic organisms such as *Anaerolineae*, *Bacteroidetes* and *Thermaceae* in *Bacteria*, and *Thermococcaceae* and the DHVEG in *Archaea*. The next dominant population was likely sulfur-oxidizing *Gammaproteobacteria*, represented by the *Sulfurivirga* sp. The hydrogenotrophic population including methanogens and hydrogen-oxidizing bacteria such as *Persephonella* sp. was apparently smaller than heterotrophic and sulfur-oxidizing populations (Figs. 1, 2). The lower abundance of hydrogenotrophic organisms in this environment is consistent with the relatively low hydrogen concentration in the venting fluids in this hydrothermal field (9).

We also noted the relatively high abundance of the *Anaerolineae* species in the microbial ecosystem that hydrogenotrophic organisms shared scare population. Previously, the relatively high abundance of *Anaerolineae* phylotypes/strains in man-made environments has been observed under methanogenic conditions, and some of the *Anaerolineae* strains from such environments have been obtained from syntrophic enrichment with methanogens (32). The observation in this microbial ecosystem suggests that such metabolic association with hydrogenotrophic organisms was not necessary for the high abundance of *Anaerolineae* species in this hydrothermal environment.

### Characterization of *Chloroflexi* strain SW7^T^

Similarity and phylogenetic analyses of the SSU rRNA gene sequence with previously isolated strains indicated that strain SW7^T^ belongs to the *Chloroflexi* class *Anaerolineae*. Strain SW7^T^ shows typical features commonly observed among the *Anaerolineae* species, such as anaerobic and fermentative metabolism and cellular morphology. Relatively low SSU rRNA gene sequence similarities (below 90%) between SW7^T^ and the *Anaerolineae* species and its distinct genomic G+C content among the *Anaerolineae* species (Table 1) indicate that strain SW7^T^ is genetically different from previously characterized species in the class *Anaerolineae*. Furthermore, the phylogenetic position of strain SW7^T^ in the SSU rRNA gene phylogenetic tree suggests that the strain represents a novel lineage in the class *Anaerolineae* (Fig. 5).

Significant physiological dissimilarities between strain SW7^T^ and *Anaerolineae* species were also noted. Optimum NaCl concentration (1.0%) for growth and inability of growth in the absence of NaCl in strain SW7^T^ showed that it is a typical marine microorganism, while NaCl inhibits the growth of other terrestrial *Anaerolineae* strains except for halophilic *T. daxensis* (Table 1). The relatively fast generation time (4.6 h) is also distinct from the long generation time (up to 100 hours) of slowly growing members within the *Anaerolineae* except for *O. apprima* (6 h). The inability to ferment using sugars as the sole carbon and energy source in strain SW7^T^ has not been reported for other *Anaerolineae* species. The fatty acid composition of the new strain, which is characterized by the relatively high abundance of unsaturated fatty acids (C_16:1_ and C_18:1_) and the existence of C_18:0_, is different from those of the previously described thermophilic strains in the *Anaerolineae* (Table 1). Considering these phylogenetic and physiological differences between strain SW7^T^ and other characterized strains in the *Anaerolineae*, we conclude here that strain SW7^T^ represents a novel species in a novel genus, for which the name *Thermomarinilinea lacunofontalis* is proposed; the type strain is SW7^T^ (=JCM 15506^T^=KCTC5908^T^).

### Description of *Thermomarinilinea* gen. nov

*Thermomarinilinea* (Ther.mo.ma.ri.ni.li’ne.a. Gr. fem. n. *therme* heat; L. adj. *marinus* of the sea; L. fem. n. *linea* line; N.L. fem. n. the “thermophilic marine line”).

Gram negative. Non-motile filamentous cells. Multicellular. Spores are not observed. Grow under obligately anaerobic conditions with fermentation. Thermophilic and neutrophilic. Growth occurs up to 65°C. NaCl is required for growth. Major fatty acids are C_16:0_ and C_18:0_. SSU rRNA gene phylogenetic analysis indicates that the genus belongs to the class *Anaerolineae* in the phylum *Chloroflexi*. Isolated from shallow sea hydrothermal environment. The type species is *Thermomarinilinea lacunofontalis*. The G + C content of genomic DNA of the type species was 59.9 mol% (HPLC).

### Description of *Thermomarinilinea lacunofontalis* sp. nov

(la.cu’no.fon’talis.. L. adj. *lacutosus* lagoonal; L. adj. *fontalis* of a spring; N.L. adj. lacunofontalis the “lagoonal spring”, as the strain was isolated from a hot spring in a coral lagoon).

Cells observed are 3.5–16.5 μm in length and 0.2 μm in width. Temperature for growth ranges between 37–65°C with optimum at 50–60°C, but unstable growth at 65°C. Growth occurs at 0.5–3.5% of NaCl concentration, and optimum is 1.0%. The growth pH range is pH 5.5–7.3, and optimum growth is observed at pH 6.0. Obligately anaerobic. Growth occurs with yeast extract, tryptone, peptone, casein, gelatin, chitin, glutamate, alanine, mannitol and citrate. The fatty acid composition was C_12:0_ (1.4%), C_16:0_ (47.0%), C_16:1_ (5.2%), C_18:0_ (37.3%) and C_18:1_ (9.1%). The G + C content of genomic DNA was 59.9 mol% (HPLC). The type strain SW7^T^ (= JCM15506^T^ = KCTC5908^T^) was isolated from Taketomi submarine hot spring shallow hydrothermal field in the southern part of Yaeyama Archipelago, Japan.

## Supplementary Material



## Figures and Tables

**Table 1:** Characteristics of cultivated species belong to the class *Anaerolineae* in the phylum *Chloroflexi*

Character	*Thermomarinilinea lacunofontalis* SW7^T^	*Anaerolinea thermophila* UNI-1^T^	*Anaerolinea thermolimosa* IMO-1^T^	*Levilinea saccharolytica* KIBI-1^T^	*Leptolinea tardivitalis* YMTK-2^T^	*Bellilinea caldifistulae* GOMI-1^T^	*Longilinea arvoryzae* KOME-1^T^	*Thermanaerothrix daxensis* GNS-1^T^	*Ornatilinea apprima* P3M-1^T^
Cell diameter#	0.2	0.2–0.3	0.3–0.4	0.4–0.5	0.15–0.2	0.2–0.4	0.4–0.6	0.2–0.3	0.3–0.7
Temperature range (°C)	37–65	50–60	42–55	25–50	25–50	45–65	30–40	50–73	–400
Optimum temperature (°C)	50–60	55	50	37–40	37	55	37	65	42–45
NaCl range (%)	0.5–3.5	<1.0	<1.5	<3.0	<1.5	≤3.0	<1.5	0–10	0–2.0
Optimum NaCl (%)	1.0	0–<0.5	0–<0.25	0–<0.25	0–<0.25	0–	0–	2.0	0.1
pH range	5.5–7.3	6.0–8.0	6.5–7.5	6.0–7.2	6.0–7.2	6.0–8.5	5.0–7.5	5.8–8.5	6.5–9.0
Optimum pH	6.0	around 7.0	around 7.0	around 7.0	around 7.0	around 7.0	around 7.0	7.0	7.5–8.0
Doubling time (h)	4.6	72 (48)*	48 (10)*	56 (56)*	50 (50)*	45 (29)*	92 (38)*	100	6
Major cellular fatty acids	C_16:0_, C_18:0_, C_18:1_	C_16:0_, C_15:0_, C_14:0_	ai-C_17:0_, i-C_15:0_, C_16:0_	C_14:0_, i-C_15:0_, C_16:0_	branched C_17:0_, C_16:0_, C_14:0_	C_16:0_, C_14:0_, i-C_15:0_	i-C_15:0_, ai-C_15:0_, C_14:0_	C_16:0_, C_18:0_, i-C_17:0_, C_20:0_, i-C_15:0_, ai-C_17:0_, C_14:0_	branched i-C_15:0_, ai-C_15:0_
DNA G+C content (mol %)	59.9	54.5	53.3	59.5	48.2	54.7	54.5	57.6	55
Utilization of:									
yeast extract	+	+	+	+	+	+	+	+	+
tryptone	+	+	−	+	+	−	−	+	N.D.
peptone	+	N.D.	+	N.D.	+	N.D.	+	−	−
starch	−	+	+	−	+	−	−	−	−
glucose	−	+	+	+	+	+	−	+	+
mannose	−	+	+	+	+	+	−	+	N.D.
galactose	−	+	+	+	+	+	−	+	−
fructose	−	+	+	+	+	+	+	+	−
arabinose	−	+	+	−	+	+	−	+	−
xylose	−	+	+	+	+	+	−	+	+
ribose	−	+	+	+	+	+	−	+	N.D.
pyruvate	−	+	+	+	+	+	−	+	−
Origin	Shallow sea hydrothermal vent	Thermophilic UASB sludge	Thermophilic UASB sludge	Mesophilic UASB sludge	Mesophilic UASB sludge	Thermophilic anaerobic sludge	Rice paddy soil	Deep terrestrial hot aquifer (60°C)	Deep terrestrial hot aquifer (47°C)
References	This study	22, 31	31, 32	31, 32	31, 32	30, 31	30, 31	6	20

# All strains show multicellular filamentous morphology

* Doubling time shown in parentheses obtained under syntrophic growth with hydrogenotrophic methanogens. Substrate utilization tests were examined in the presence of yeast extract.

+, positive; −, negative; N.D., not determined
