# Supplementary material for: Isolation and Characterization of a Thermophilic, Obligately Anaerobic and Heterotrophic Marine Chloroflexi Bacterium from a Chloroflexi-dominated Microbial Community Associated with a Japanese Shallow Hydrothermal System, and Proposal for Thermomarinilinea lacunofontalis gen. nov., sp. nov
Source: Microbes Environ. 2013 May 11;28(2):228–35. doi: 10.1264/jsme2.ME12193 (PMC4070665; doi:10.1264/jsme2.ME12193)
Supplement: Supplementary file 1 [file 28_228_s1.pdf]

Supplementary text

Materials and methods

Composition of media used in this study

MJYPS medium (Takai et al. 2000)

Modified MJ synthetic seawater with yeast extract (0.1 %, w/v), trypticase peptone (0.1 %), elemental sulfur (0.3 %), resazurin (0.0001 %), and neutralized Na_2S $9H_2O$ (final 0.05 %). Head space gas was N_2 (100 kPa).

MMJ medium (Takai et al. 2002)

Modified MJ synthetic seawater with vitamin mixture (0.1 %, v/v) (Balch et al. 1979), resazurin (0.0001 %), $NaHCO_3$ (0.2 %), cysteine hydrochloride (0.05 %) and 0.5 g Na_2S $9H_2O$ 0.05 %). Head space gas was a mixture of 80% H_2 : 20% CO_2 (200 kPa).

MMJSO medium (Nunoura et al. 2007b)

Modified MJ synthetic seawater supplemented with yeast extract (0.02 %, w/v), sodium lactate (0.05 %), sodium pyruvate (0.05 %), sodium ascorbate (0.05 %), $NaHCO_3$ (0.1 %), Na_2SO_4 (0.2 %, w/v, added to MJ synthetic seawater) and resazurin (0.0001 %). The pH was adjusted to 7. A gas mixture of 80% H_2 : 20% CO_2 (200 kPa) was used for head-space gas.

MMJS medium (Nunoura et al. 2008)

Modified MJ synthetic seawater with vitamin mixture (0.1 %, v/v) (Balch et al. 1979), resazurin (0.0001 %), $NaHCO_3$ (0.2 %), elemental sulfur (3%) and 0.5 g Na_2S $9H_2O$ 0.05 %). A gas mixture of 80% H_2 : 20% CO_2 (200 kPa) was used for head-space gas.

MMJHS medium (Takai et al. 2003)

Modified MJ synthetic seawater with vitamin mixture (0.1 %, v/v) (Balch et al. 1979), sodium thiosulfate (0.1%) and elemental sulfur (0.3%), but with sodium nitrate (0.1%) in the absence of headspace O_2. Three types of head space gases of 80%

H_2 and 20% CO_2 (200 kPa), and 79% H_2, 20% CO_2 and 1% O_2 (200 kPa) were used in this study.

MMJYPS medium (Nunoura et al. 2007a)

Modified MJ synthetic seawater, yeast extract (0.1%), tryptone peptone (0.1%), sulfur (0.3%), and resazurin (0.0001 %), at a pH adjusted to around 5.5. Head space gas was a mixture of 80% H_2 : 20% CO_2 (200 kPa)

MJY medium (Nunoura et al. 2010)

Modified MJ synthetic seawater with yeast extract (0.1 %, w/v), resazurin (0.0001 %), and neutralized Na_2S $9H_2O$ (final 0.05 %). Head space gas was N_2 (100 kPa).

Supplementary figures

(a) (b)

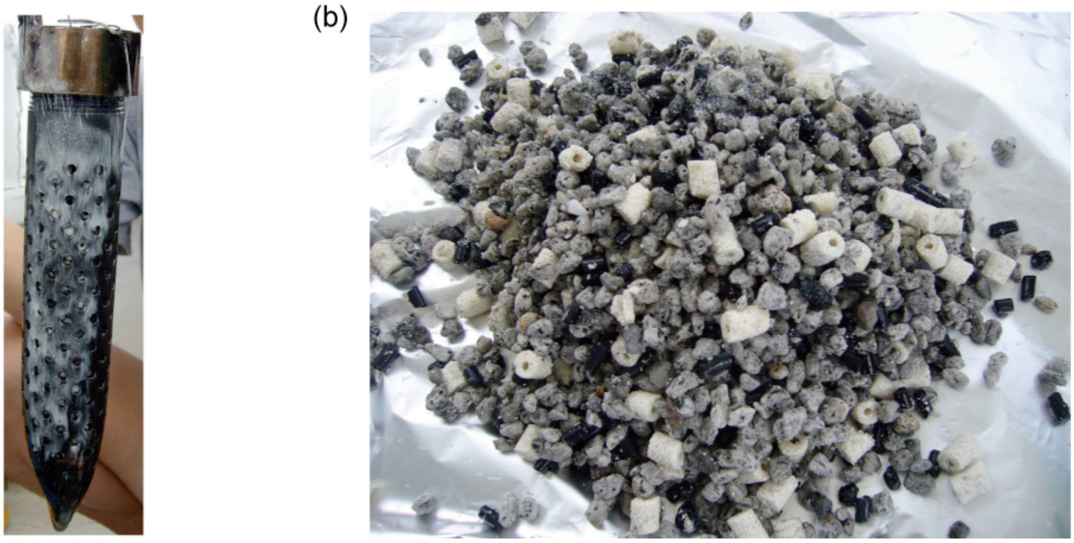

Figure S1. (a) Appearance of *in situ* colonization system (ISCS) after 5 days of incubation in a hydrothermal vent emission. Inner diameter and inner length of the ISCS were 45 and 250 mm, respectively. Filamentous white microbial mat formations were observed. (b) Pumiceous stuffing settled in the ISCS after the 5 day of incubations. Outer diameter and length of the white rings were 6 and 9 mm, respectively.

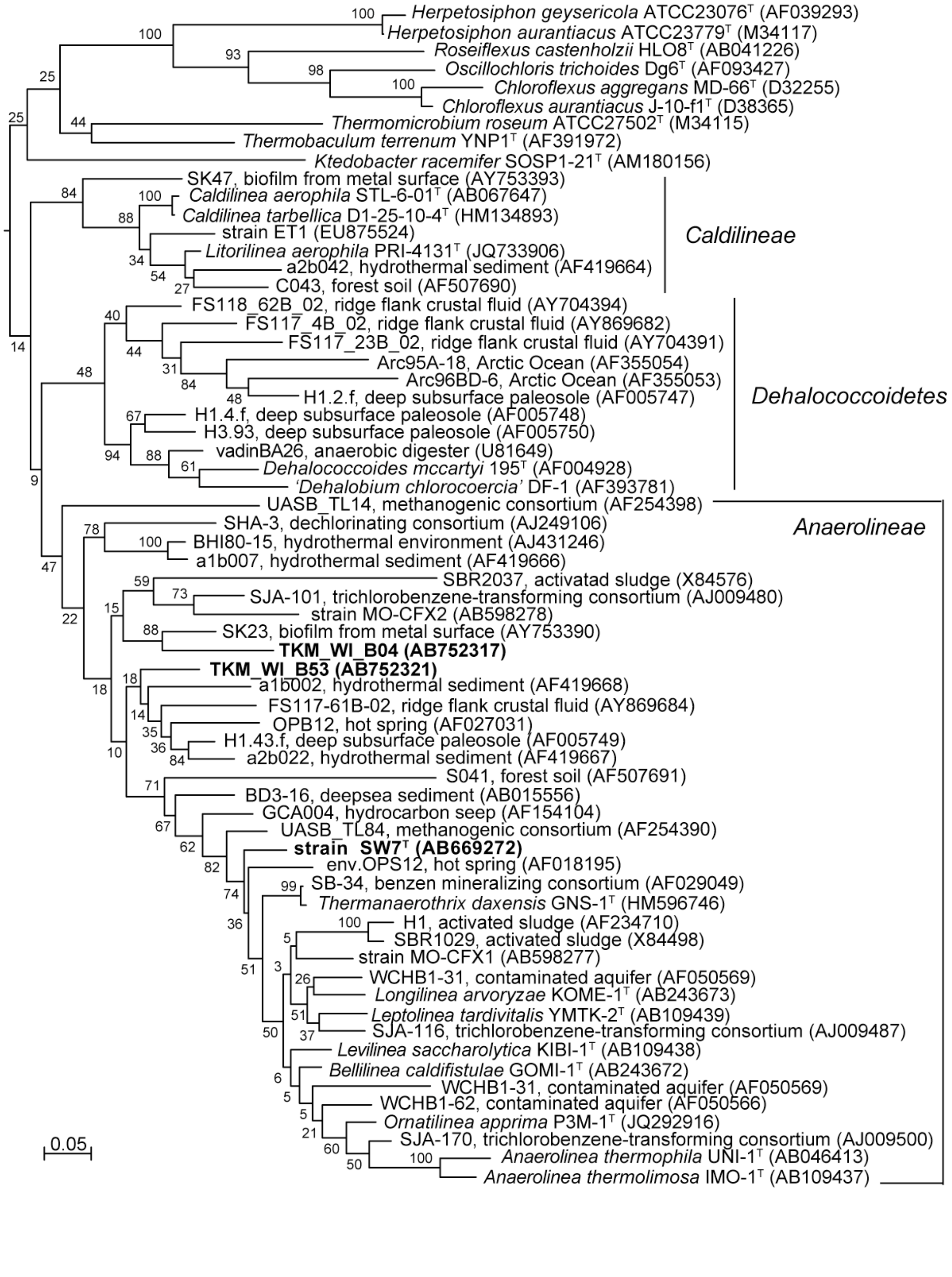

Figure S2. Phylogenetic tree of the phylum *Chloroflexi* based on SSU rRNA gene sequence by PhyML using 707 homologous sequence positions. Bold indicate the sequences obtained in this study. Numbers indicate bootstrap values from 100 trials. Numbers in parentheses are GenBank/EMBL/DDBJ accession number. Bar indicates 5 substitutions per 100 nucleotides. *Desulfovibrio vulgaris* DSM644 (M34399) was used for an out-group.

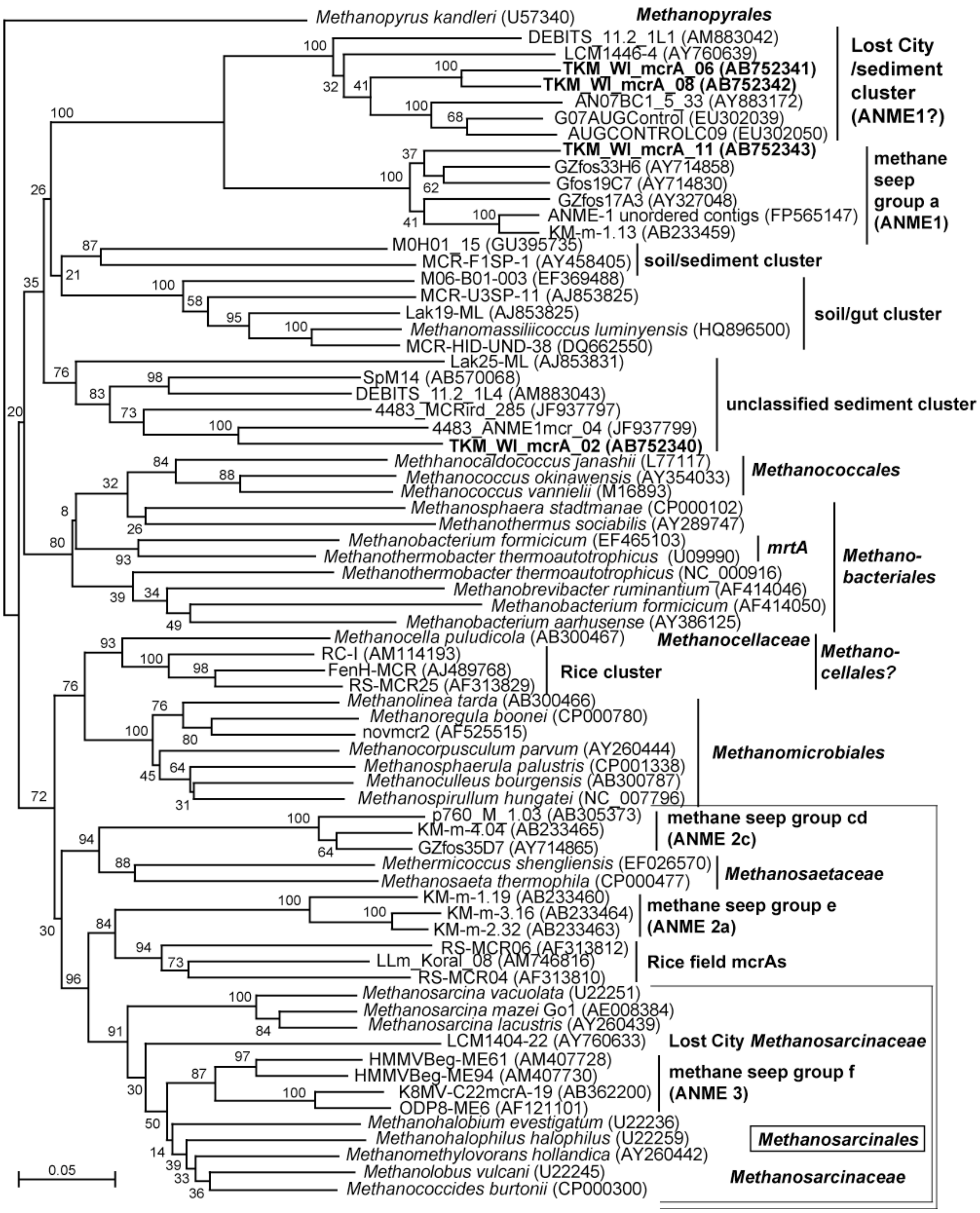

Figure S3. Phylogenetic tree of the *mcrA* nucleotide sequences by neighbor-joining method. Bold indicate the sequences obtained in this study. Numbers indicate bootstrap values from 100 trials. Numbers in parentheses are GenBank/EMBL/DDBJ accession number. Bar indicates 5 substitutions per 100 nucleotides.

Table S1. Sequences of primers and probes, and PCR conditions used in this study.

Target gene	Primer	Sequence (5' 3')	PCR condition	Reference
**Clone analyses for environmental DNA**				
archaeal SSU rRNA gene	A21F	TTCCGGTTGATCCYGCCGGA	96°C for 1min, 30 x (96°C 25s, 50°C 45s,72°C 90s)	DeLong 1992
	U907R	CCCGTCAATTCCTTTGAGTTT		
bacterial SSU rRNA gene	B27F	AGAGTTTGATCCTGGCTCAG	96°C for 1min, 25 x (96°C 25s, 53°C 45s,72°C 90s)	Lane 1985
	U907R	CCCGTCAATTCCTTTGAGTTT		
mcrA	ME3MF	ATGTCNGGTGGHGTMGGSTTYAC	94 °C for 2 min, 35 x (94°C for 40 s, 52°C for 30 s, and 72°C for 1 min)	Hales et al. 1996, Nunoura et al. 2008
	ME2r'	TCATBGCRTAGTTDGGRTAGT		
**Quantitative PCR**				
prokaryotic SSU rRNA gene	Uni340F	CCTACGGGRBGCASCAG		
	Uni516F	TGYCAGCMGCCGCGGTAAHACVNRS	96°C for 1min, 50 x (96°C 25s, 57°C 6min)	Takai & Horikoshi 2000
	Uni806R	GGACTACNNGGGTATCTAAT		
archaeal SSU rRNA gene	Arch349F	GYGCASCAGKCGMGAAW		
	Arch516F	TGYCAGCCGCCGCGGTAAHACCVGC	96°C for 1min, 50 x (96°C 25s, 59°C 6min)	Takai & Horikoshi 2000
	Arch806R	GGACTACVSGGGTATCTAAT		
mcrA	ME3MF	ATGTCNGGTGGHGTMGGSTTYAC	94 °C for 2 min, 40 x (94°C for 40 s, 52°C for 30 s, and 72°C for 1 min)	Hales et al. 1996, Nunoura et al. 2008
	ME2r'	TCATBGCRTAGTTDGGRTAGT		
**Amplification for isolates**				
archaeal SSU rRNA gene	A21F	TTCCGGTTGATCCYGCCGGA	96°C for 1min, 30 x (96°C 25s, 50°C 45s,72°C 2min)	DeLong 1992
	U1492R	ASGGNTACCTTGTTACGACTT		
bacterial SSU rRNA gene	B27F	AGAGTTTGATCCTGGCTCAG	96°C for 1min, 25 x (96°C 25s, 53°C 45s,72°C 2min)	Lane 1985
	U1492R	ASGGNTACCTTGTTACGACTT		

Table S2. Bacterial and archaeal SSU rRNA gene community structures associated with ISCS deployed in the main vent of the Taketomi hydrothermal field.

(A) *Bacteria*

Taxon	Phylotype	Number of sequences	Accession number
Chloroflexi	TKM_WI_B04	13	AB752317
	TKM_WI_B53	1	AB752321
Deltaproteobacteria	TKM_WI_B50	1	AB752319
	TKM_WI_B60	6	AB752322
	TKM_WI_B76	1	AB752328
Bacteroidetes	TKM_WI_B68	4	AB752326
	TKM_WI_B75	1	AB752327
Deferribacterales	TKM_WI_B62	2	AB752323
Caldithrix group	TKM_WI_B63	1	AB752324
	TKM_WI_B65	1	AB752325
Thermaceae	TKM_WI_B08	3	AB752318
Gammaproetobacteria	TKM_WI_B52	1	AB752320
Total		35	

(B) *Archaea*

Taxon/division	Phylotype	Number of sequences	Accession number
Euryarchaeota			
Thermococcaceae	TKM_WI_A50	11	AB752331
	TKM_WI_A70	1	AB752337
DHVEG	TKM_WI_A52	11	AB752333
	TKM_WI_A77	1	AB752338
Lost City *Methanosarcinales*	TKM_WI_A14	2	AB752329
MEG	TKM_WI_A51	1	AB752332
SAGMEG	TKM_WI_A59	1	AB752334
DHVE8	TKM_WI_A78	1	AB752339
Other *Archaea*			
DSAG	TKM_WI_A49	3	AB752330
	TKM_WI_A64	1	AB752335
MCG	TKM_WI_A68	2	AB752336
Total		35	

Table S3. *mcrA* gene community structures associated with ISCS deployed in the main vent of the Taketomi hydrothermal field.

Phylogroup	Phylotype	Number of sequences	Accession number
ANME I	TKM_WI_mcrA_11	8	AB752343
ANME I-like cluster	TKM_WI_mcrA_06	3	AB752341
	TKM_WI_mcrA_08	7	AB752342
unclassified sediment cluster	TKM_WI_mcrA_02	7	AB752340
Total		25	

Table S4 . Viable numbers of representative strains obtained by serial dilution count cultivation analysis from the ISCS deployed in the main vent of the Taketomi hydrothermal field.

Medium	Incubation temperature		
	85 °C	70 °C	55 °C
MJYPS	1×10^6<br>I.N.O. (*Thermococcaceae*-like)	N.T	1×10^7<br>*Thermomarinilinea* strain SW7^T<br>(AB669272)<br>*Thermococcus* sp. TKM-55-W7-A<br>(AB752314)
MMJ	N.T.	-	N.T.
MMJSO	1×10<br>*Thermotoga* sp. TKM-83SO-W1<br>(AB752310)	1×10^2<br>*Thermotoga* sp. TKM-70SO-W2<br>(AB752311)	1×10^2<br>*Caminibacillus* sp. TKM-55SO-W2<br>(AB752312)
MMJS	-	-	N.T.
MJYHS	-	1×10<br>I. N.O.	-
MJYHS O2-1%		1×10^2<br>*Persephonella* sp. TKM-70-1-W2<br>(AB752315)	1×10^7<br>*Sulfurivirga* sp. TKM-55-1-W7<br>(AB752316)
MMJYPS	-	1×10^4<br>I.N.O. (*Deferribacter*-like)	1×10^4<br>*Deferribacter* sp. TKM 55H-W4<br>(AB752313)

I.N.O, isolate not obtained after dilution counting.
N.T., not tested.
-, no growth.

References in the supplementary materials

Balch, W.E., G.E. Fox, L.J. Magrum, C.R. Woese, and R.S. Wolfe. 1979. Methanogens: reevaluation of a unique biological group. Microbiol. Rev. 43:260-296.

DeLong, E.F. 1992. Archaea in coastal marine environments. Proc. Natl. Acad. Sci. USA. 89:5685–5689.

Hales, B.A., C. Edwards, D.A. Ritchie, G. Hall, R.W. Pickup, and J.R. Saunders 1996. Isolation and identification of methanogen-specific DNA from blanket bog peat by PCR amplification and sequence analysis. Appl. Environ. Microbiol. 62:668–675.

Lane, D.J. 1985. 16S-23S rRNA sequencing, p. 115-175. *In* E. Stackebrandt, and M. Goodfellow (ed.), Techniques in Bacterial Systematics. Wiley, Chichester.

Nunoura, T., H. Oida, J. Miyazaki, A. Miyashita, H. Imachi, and K. Takai. 2008. Quantification of *mcrA* by fluorescent PCR in methanogenic and anaerobic methanotrophic microbial communities. FEMS Microbiol. Ecol. 64:240-247.

Nunoura, T., H. Oida, M. Miyazaki, and Y. Suzuki 2008. *Thermosulfidibacter takaii* gen. nov. sp. nov. a thermophilic hydrogen oxidizing, sulfur reducing bacterium isolated from a deep-sea hydrothermal field, Southern Okinawa Trough within the phylum

Aquificae. Int J Syst Evol Microbiol. 58: 659-665.

Nunoura, T., H. Oida, M. Miyazaki, Y. Suzuki, K. Takai, and K. Horikoshi. 2007a. *Marinitoga okinawensis* sp. nov. a novel thermophilic and anaerobic heterotroph isolated from a deep-sea hydrothermal field, Southern Okinawa Trough. Int. J. Syst. Evol. Microbiol. 57: 467-471.

Nunoura, T., H. Oida, M. Miyazaki, Y. Suzuki, K. Takai, and K. Horikoshi. 2007b. *Desulfothermus okinawensis* sp. nov. a thermophilic and heterotrophic sulfate-reducing bacterium isolated from a deep-sea hydrothermal field. Int. J. Syst. Evol. Microbiol. 57:2360-2364

Nunoura, T., M. Hirai, H. Imachi, M. Miyazaki, H. Makita, H. Hirayama, Y. Furushima, H. Yamamoto, and K. Takai. 2010. *Kosmotoga arenicorallina* sp. nov. a thermophilic and obligately anaerobic heterotroph isolated from a shallow hydrothermal system occurring within a coral reef, southern part of the Yaeyama Archipelago, Japan, reclassification of *Thermococcoides shengliensis* as *Kosmotoga shengliensis* comb. nov., and emended description of the genus *Kosmotoga*. Arch. Microbiol. 192:811-819.

Takai, K. and K. Horikoshi. 2000. Rapid detection and quantification of members of the archaeal community by quantitative PCR using fluorogenic probes. Appl. Environ. Microbiol. 66:5066-5072.

Takai, K., F. Inagaki, S. Nakagawa, H. Hirayama, T. Nunoura, Y. Sako, K.H. Nealson, and K. Horikoshi 2003. Isolation and phylogenetic diversity of members of previously uncultivated epsilon-Proteobacteria in deep-sea hydrothermal fields. FEMS Microbiol. Lett. 218:167-174.

Takai, K., A. Inoue, and K. Horikoshi 2002. *Methanothermococcus okinawensis* sp. nov., a thermophilic, methane-producing archaeon isolated from a Western Pacific deep-sea hydrothermal vent system. Int. J. Syst. Evol. Microbiol. 52: 1089-1095.

Takai, K., A. Sugai, T. Itoh, and K. Horikoshi. 2000. *Palaeococcus ferrophilus* gen. nov., sp. nov., a barophilic, hyperthermophilic archaeon from a deep-sea hydrothermal vent chimney. Int. J. Syst. Evol. Microbiol. 50:489–500.
